# Supplementary material for: Are carbon emissions trading and green financial instruments synergistic? -Comprehensive quantitative research based on content analysis
Source: PLoS One. 2024 Mar 7;19(3):e0298601. doi: 10.1371/journal.pone.0298601 (PMC10919601; doi:10.1371/journal.pone.0298601)
Supplement: S1 Data — (ZIP) [file pone.0298601.s001.zip › Data/地级市Z_F工作报告.zip!/┴╔─■/▒╛╧¬╩╨/└┤╫╘╣1⁄2╓┌║┼í╛┬φ┐╦╩2╛▌═°í┐_2017.docx]

各位代表：

　　我代表本溪市人民政府向大会报告工作，请予审议，并请市政协委员和各位列席会议的同志提出意见和建议。

　　一、2016年工作回顾

　　2016年，市政府始终坚持五大发展理念不动摇，以推进供给侧结构性改革为主线，抢抓新一轮振兴发展机遇有新作为，聚焦五大产业、项目建设、创新驱动增强发展新动能，防范风险、保障和改善民生取得显著成效，保持了经济社会平稳健康发展态势，夯实了全市“十三五”稳中有进、稳中向好的发展基础。

　　在应对增长速度换档期、结构调整阵痛期、前期刺激政策消化期“三期叠加”挑战、统筹安排做实数据背景下，全年固定资产投资235亿元；一般公共预算收入55.6亿元；社会消费品零售总额377.4亿元；规模以上工业增加值下降33.5%；出口总额18.9亿美元；实际直接利用外资6076万美元；金融机构本外币贷款余额1076.9亿元；城镇常住居民人均可支配收入29137元；农村常住居民人均可支配收入13574元；居民消费价格指数（CPI）累计增长1.2%。

　　一年来，市政府主要做了以下六方面工作：

　　（一）着力完善体制机制，改革开放实现新突破

　　深化推进投融资体制改革。组建了旅游、水务、文化等11家控股公司并挂牌运营，带动4家事业单位改制，注入资产1.06亿元。推进设立五大产业发展基金，申报药都健康医疗、三生生物医药健康、现代农业3支产业（创业）投资基金，获省引导基金支持4.5亿元，基金数量及金额均排全省第二位。

　　深入推进行政管理体制改革。共取消下放调整行政职权306项，清理规范中介服务事项61项。成立本溪市行政审批服务局。大力推进“五证合一”等登记制度改革,全市新登记市场主体10265户。实施不动产统一登记制度走在全省前列，全年发放不动产权证书18200本、不动产登记证明9126张。顺利完成公务用车制度改革。

　　稳妥推进财税体制改革。积极推进财政绩效、全口径预算管理，全面推动预决算公开。严控支出实现了保工资、保运转、保民生目标。盘活财政存量资金、全面推开营改增、促进国地税深度融合等工作顺利推进。

　　平稳推进农村土地制度、林业改革。完成农村土地确权颁证登记面积53万亩，全市土地流转总面积6.3万亩。完成国有林场改革实施方案，枫林谷国有林场转型经验得到国家肯定，桓仁县集体林“三权分置”取得新进展。东北林业产权交易中心实现全省50个县级分中心互联互通。

　　持续推进职业教育体制改革。建设11个省级产业集群示范专业、16个省级创业型实训基地，引厂入校和校企融合实现突破。

　　扎实推进国资国企改革。出台了经营性国有资产监督管理办法等文件，推进了61个党政机关与所办122个企业脱钩、破产及僵尸企业处置，扎实开展驻溪央企“三供一业”分离移交等工作。

　　不断推进对外开放。支持本钢、大石药业等重点出口企业融入“一带一路”，扩大产能输出合作。高新区3家药企“走出去”实现在美投资。围绕五大产业，面向京津冀、长三角、珠三角开展招商活动1405次，洽谈项目561个，福耀高端浮法玻璃、万达广场、康力顺势产业园等重大项目签约落地，签约额340亿元。

　　（二）着力推进结构调整，经济发展质量效益实现新提升

　　打好“三去一降一补”攻坚战。关闭煤矿16家、化解产能69万吨，提前超额完成省定任务。利用春秋两季房交会、棚户区改造去库存，棚改货币化安置比例达89%，全年商品房销售面积、销售额分别实现72.7万平方米、32亿元。建立债务风险预警系统，通过规范“借用还”一体化管理、安排到期政府债务纳入财政收支预算等举措去杠杆，有效防范政府债务风险。实行矿产资源税从价计征，解决直供电3.9亿千瓦时，为企业降成本1.13亿元。注重扩大有效投入、提升民生保障水平，补齐发展短板。

　　深入推进“三都”建设。绿色钢都建设取得成效。落实“大材料”产业规划，服务本钢稳定生产，基本解决了长期制约发展的本钢冷轧高强钢项目铁路专用线等突出问题。本钢积极调结构、提品质、拓市场，实现扭亏增盈。本钢冷轧高强钢、华岳精工锻钢曲轴等一批项目竣工投产，金桥焊材、银龙预应力、东铄新材料等重点钢铁深加工企业产值成倍增长。中国药都建设成果丰硕。突出“大健康”产业发展重点，引进日本卫材等国际知名企业入驻高新区。中海康药业、迈迪生物和晟麦实业3家企业在新三板挂牌上市，实现我市新三板上市企业零突破。销售收入超亿元企业13家、单品种产品8个。医药企业销售收入增长11%，税收增长27.2%，高于全市平均水平37个百分点。上海绿谷、上药集团等52个亿元以上项目开复工，新签约项目25个、合同投资额68.4亿元。沈阳药科大学实现整体搬迁，1万余名师生全面入驻，辽宁中医药大学本溪校区、辽宁医药职业学院、东方剑桥国际学校一期工程主体封顶。枫叶之都建设取得进展。促进“大旅游”产业加快发展，出台了支持旅游业改革发展政策意见，在全省率先启动本溪水洞国有景区管理体制改革。本溪县、桓仁县被评为“全国森林旅游示范县”。大石湖、枫林谷晋升为国家4A级旅游景区，温泉寺和汤沟温泉度假区、枫林谷房车自驾营地等项目建设稳步推进。培育了同江峪、甬子峪特色民宿及“五朵金花”等消费热点。成功举办桓仁首届世界太极养生文化高峰论坛等活动。以CBA赛事激活冬季旅游。全年旅游收入、接待游客数量分别增长14.5%和13.5%。打造“三都五城”升级版，编制五大产业发展纲要，将一张好的蓝图真正绘到底。

　　全力加快项目升级年建设。成立重大项目建设工作领导小组，加强政府投资建设项目管理。推进本溪热电、思山岭铁矿、观音阁水库输水工程等重点项目实现开复工，亿元以上项目开复工率达96%。抢抓国家振兴发展政策机遇，助推项目建设。我市成为中德生物技术和医药产业重点合作区域。生物医药集群列入国家新兴产业三年行动计划，享受连续3年、每年2亿元专项基金扶持。辽中环高速本溪至铁岭段、本桓高速和轨道交通项目列入东北振兴三年滚动实施方案。棚户区改造获国开行、农发行贷款24亿元，国家专项建设基金获批7个项目、3.63亿元, 本钢成功申请发行非公开可交换公司债券40亿元。南芬独立工矿区改造搬迁、桓仁镇投融资模式创新小城镇建设等获国家专项资金支持。本溪县获批国家新型城镇化综合试点，我市成为全省唯一的深化中小城市PPP创新工作试点，成功承办了高层次推进PPP项目工作会议。

　　进一步巩固产业协调发展势头。工业经济平稳运行。积极解决规上企业土地、供电等问题176项，协调本钢签订本地铁精粉供货合同252万吨，促进停产半停产企业恢复生产，带动工业用电量、货运量等指标增速回升。组织开展了经济景气监测。全市抓工业经济发展的氛围更加浓厚。服务业发展态势良好。华联物流配送中心、东北参茸城等商贸流通项目投入运营，长江农副产品智能物流交易中心加快主体施工。高新区国家电子商务示范基地建设有序推进，本溪首家保税仓暨电商平台封仓运行。营口银行、锦州银行本溪分行等正式营业，市商业银行增资扩股加快推进，本溪县、南芬农信社完成改制农商行申报准备工作。新增社会融资总量突破210亿元。农业稳步发展。调减玉米种植面积2.9万亩，优化了种植结构。全年粮食产量30万吨。本溪县刺五加及制品通过国家生态原产地保护产品评定，实现辽宁地区零突破，刺五加深加工龙头企业达6家。桓仁县葡萄原料基地面积发展到7万亩。北药基地建设完成耕地中药材种植12.9万亩、经济林基地5.1万亩，启动澳地森3000亩国家级中药材种苗繁育基地建设。开展农业普查摸清“三农”家底。完成民生水利工程400余项。实施农村信息进村入户工程，建立益农信息社192个，农村信息覆盖率达80%以上。

　　（三）着力鼓励创新创业，经济发展动力实现新跃升

　　持续完善技术创新体系。构建了集112家生产经营单位、50家科研机构、6所医药类高等院校于一体的协同创新体系。本钢汽车用钢开发与应用实验室升级为国家研发平台，上海绿谷糖类药物实验室获批组建省级重点实验室，以企业为主体、产学研用相结合的技术创新体系加速形成。

　　加快科技成果转化。高新区八大公共服务平台提供检测、分析和技术开发服务近8000项，组建了高新区大型科学实验仪器共享联盟，桓仁葡萄酒等产业技术创新战略联盟申报省级试点，带动科技成果共享、快速转化。

　　强力推进智能制造。制定《本溪市制造+智造强市建设行动纲要》，实施147个制造业、30个智能制造项目建设。同仁堂药业进入省智能制造标杆企业评选阶段。提速“大数据”产业发展，加大智慧城市建设力度，成为省级三网融合试点城市，整合资金1.1亿元建成云数据中心等项目，70家“智慧社区”、“智慧党建”系统投入运营。

　　创新机制招才引智。出台了《本溪市机关事业单位高层次急需紧缺人才引进办法》，引进31个高水平人才智力团队，建成华日氟高分子材料、龙宝集团北药院士工作站。

　　推进“双创”助力中小微企业发展。编制“双创”五年行动规划实施方案，建设各类人才创新创业基地39个，中国药都创新园众创空间被省评为第一批“云启众创”。开展中小微企业培训3000余人次、银企对接活动20余次，发放创业担保贷款6046万元。

　　（四）着力保障和改善民生，人民群众生活质量实现新提高

　　确保企业退休人员养老金按时足额发放。加强养老保险费征缴清欠，强化监管，通过建立市、县（区）共担养老保险基金收支缺口等举措，保证了养老金按时足额发放。

　　保持就业形势稳定。切实加强就业援助，帮助全市2000余名就业困难人员实现就业，继续保持零就业家庭动态为零。实现城镇新增就业2.58万人，登记失业率控制在3.3%。

　　有序推进精准扶贫。落实“六个精准”要求，8家省直单位、64家市直单位、72个驻村工作队与贫困村结成帮扶对子，两县整合资金1.16亿元用于精准脱贫，全面完成年度脱贫任务。

　　稳步提升社会保障水平。企业退休人员基本养老金月人均增加143元、达到2232元。提高月最低工资标准，市区由1050元调整到1320元，两县由900元调整到1200元。提高城镇居民医保财政补助以及城乡低保、农村五保标准。建立了困难残疾人生活补贴和重度残疾人护理补贴制度，受益2万余人。完成贫困残疾人辅具适配2382件，家庭无障碍改造706户。

　　加快发展教育事业。普惠性幼儿园覆盖率达66%。本溪县、桓仁县、溪湖区、南芬区和高新区通过国家义务教育发展基本均衡县（区）认定，跻身全省前列。太子河新城实验学校完成规划设计，全市百余所学校办学环境、教育装备全面改善。全市高考录取率、一本上线率继续在全省领先。辽宁科技学院获批建设中德教育合作基地。中国联合国教科文组织全国委员会将我市评为教育信息化工作试点单位。

　　协同发展文化事业和文化产业。群众文化“双进双建”活动通过国家级公共文化服务示范项目验收。举办全市文化艺术节和群众文化节，开展群众文化活动400余场。本溪文艺家之家正式启用。发展特色文化产业，完成辽砚研发和检测中心、铁刹山农民工笔画艺术中心扩建工程，启动本溪湖工业遗址博览园建设。实现市新华书店转型改制。

　　创新发展卫生、体育等事业。市、县公立医院综合改革稳步推进，走在全省前列。组建了市中心医院等3个“医联体”，分级诊疗制度逐步建立，家庭医生签约服务试点全面启动。推进县、乡、村一体化管理试点工作。实现基本药物制度行政村全覆盖。在全国地级市中率先制定大健康产业规划。构建“一县一品牌、一区一特色”体育产业新格局，明山区申报国家级体育产业示范基地通过省初审。本桓公路成为“辽宁省公路自行车训练基地”。19个社区多功能运动场等设施建成投入使用。同时，人防、外事、侨务、对台、民族、宗教、档案、史志、气象等工作取得积极进展，妇女儿童等事业得到新发展。

　　始终保持社会和谐稳定。推进立体化社会治安防控体系建设，构建“全警大巡防”模式。深入开展打击“盗抢骗”等专项行动，依法处理“7.04”非法盗采案件。全面深化公安改革，在放宽落户政策等方面取得显著成效。严格落实安全生产责任制，确保安全度汛。建立市、县（区）领导信访接待日制度，开展党政领导化解信访难题“百日攻坚”专项行动，集中化解信访案件1247件、占比达90%。实现“六五”全国法制宣传教育先进城市“四连冠”、全国双拥模范城“八连冠”目标。

　　顺利完成民生实事任务。一是24个省级贫困村、10194名贫困人口脱贫任务实现销号。二是30个棚户区改造项目开工，开工总套数10160套、完成任务的103.4%。完成569户农村危房改造任务。三是实施“暖房子”工程438.6万平方米，完成任务的146.2%。完成住宅区环境整治58.1万平方米。四是育龙路至本桓线、千金路至丹霍线城市出口建设进展顺利，郑家大桥竣工通车。五是完成街巷路改造39条、改造人行道板3.69万平方米。六是新增停车泊位2155个。七是本宽线北大岭隧道及引线旅游环线工程已开工建设，旅游景点新建、改扩建公厕40处。八是提前超额完成主城区25所中小学体育场馆面向市民开放任务。九是市中心医院太子河新城综合病房楼、市失能半失能老人康复养护中心项目完成主体施工，新建10个社区养老服务中心。十是完成56万张市民卡发放工作，我市公交线路全面开通市民卡刷卡乘车功能。

　　（五）着力统筹推进城乡建设，区域发展实现新跨越

　　全面开展市容环境综合整治行动。治理城市“五乱”14.8万处，拆除私搭乱建4300余处，取缔流动烧烤1300余处，48条市区主次干路、望溪公园等地补植树木8000余株，实施百日攻坚交通整治取得明显成效，市容环境更加整洁有序。谋划开展主城区环境综合整治行动，计划用两年时间完成“暖房子”、居民住宅小区环境、街巷路及交通整治工程，并在媒体公示承诺书、实施方案及监督电话，确保将好事办实、办到位。

　　加速主城区向北拓展。太子河新城新型城镇化、地下综合管廊及“新溪湖·印象”等项目建设稳步推进。实施威宁村整体搬迁，威宁大街建成通车，威宁保障房及梁山大街、鑫源路等项目建设有序推进。

　　加快构建“大交通”布局。完成沈环线三家子至卧龙段改建等主体工程。完成本桓线等国家、省干线大中修116.8公里、农村公路40.4公里。鲍家两洞桥改造工程进展顺利。丹阜高速石桥子至本溪出口太阳能路灯亮化工程投入使用。推进了田桓铁路和辽中环高速本溪段拆迁、本桓高速选线等前期工作。

　　积极创建生态文明建设示范市。不断完善“大生态”产业体系，编制创建生态文明建设示范市规划，本溪县、桓仁县获批国家重点生态功能区。深入推进“五城”建设，“创模”“创卫”基本完成重点项目任务。大力实施“青山、碧水、蓝天”工程，完成营造林17万亩。积极应对恶劣天气，控制污染物排放，完成取缔禁燃区内230个10吨以下燃煤锅炉省定任务，并扩大成果多取缔416个；淘汰黄标车及老旧车辆19747台，全年城区环境空气质量达标天数占比88.2%。提高太子河地表水质量，饮用水水源水质达标率100%，国控、省控断面水质符合国家标准要求。开展文明祭祀宣传活动，殡仪服务中心搬迁项目办公楼、综合服务楼等主体竣工。

　　扎实推进宜居乡村建设。完成村内道路硬化413.9公里，饮水改造58个村、受益7.64万人。建设卫生厕所2179个，清理垃圾4.1万吨。城区所辖13个涉农街道、84个村庄全面完成治理任务。

　　（六）着力加强政府自身建设，施政能力和服务水平实现新提升

　　切实改进工作作风。把学习贯彻十八届六中全会精神、习近平总书记系列重要讲话精神作为首要政治任务，扎实开展“两学一做”学习教育，打牢振兴发展的思想政治基础。推进阳光政府建设，加大政务公开力度，向社会公开各类信息2.8万余条。继续坚持“开门写报告”，征集民生实事意见建议318条。

　　大力推进依法行政。坚决落实市委决策部署，坚持每季度向市委汇报工作。自觉接受市人大及其常委会的法律监督、工作监督和市政协的民主监督。553件省、市建议提案全部按期办复，530件市本级建议提案解决率达71.9%。制订规范性文件17件。

　　强化廉洁从政。严守政治纪律和政治规矩，严格遵守中央八项规定，认真履行“一岗双责”。在高新区率先开展驻区企业和高校评议市政府涉企部门服务试点，努力构建亲、清新型政商关系，切实打造良好营商环境。

　　各位代表，2016年本溪经济社会发展取得的成绩，是在严峻经济形势下，市政府始终坚持在市委的正确领导下，在市人大、市政协的监督支持下，在全市上下的共同努力下，坚定发展信心、保持战略定力，迎难而上、创新实干取得的。成绩来之不易、难能可贵。在此，我代表市政府，向全市人民、社会各界，向支持和参与本溪发展建设的朋友们，表示衷心的感谢和崇高的敬意！

　　在总结工作的同时，还需要我们正视存在的问题。

　　一是工业经济企稳基础还不稳固。我市产业结构偏重矛盾仍然突出，特别是采选企业占比较大，多数企业处于停产半停产状态，生物医药等新兴产业虽然快速发展，但总量较小尚不能弥补传统产业回落份额，面临巨大转型升级压力。同时，钢铁、煤炭、水泥等行业仍处于化解过剩产能阶段，由于房地产消费不旺，原材料价格虽有所回升但仍在低位运行，工业企稳基础需进一步巩固。

　　二是投资动力不足。全市项目数量总体下降，纳入统计口径固投项目357个，下降32.6%，民间投资、房地产投资下降幅度较大，新产业、新业态项目少的问题仍然突出。其中，民间投资下降65.3%，由于民间投资占全市比重接近60%，对全市固投增长影响较大；旅游、健康养老等新兴产业投资占比仅为20%，带动长远发展动力不足。

　　三是财政收支矛盾十分突出。我市地方财力薄弱，保工资、保运转、保民生已面临巨大压力，还需妥善解决历史遗留问题、化解政府债务风险、解决企业退休人员养老基金缺口逐年增加等突出问题，加之我市财政供养人口过多，财政收支矛盾日益凸显，防风险任务繁重而艰巨。

　　四是部分干部工作不到位。个别干部思想不够解放，不能适应新常态工作要求，创新能力不足，且执行力不强、深入基层不多、群众观念淡薄，导致办事效率低、服务质量差、重点工作无成效，究其原因是缺乏坚定的政治信念、忠诚担当的精神和干事创业的热情。

　　对于这些问题，我们必须要深刻反思，查找差距和不足，以更大的决心、勇气推进整改解决。

　　二、2017年主要任务

　　2017年是实施“十三五”规划的重要一年，是推动供给侧结构性改革的深化之年，是全面落实新一轮振兴政策的关键一年。今年政府工作的总体要求是：认真贯彻落实十八届六中全会、中央和省经济工作会议及省、市党代会精神，牢固树立新发展理念，落实“四个着力”要求，全力抢抓新一轮振兴发展机遇，坚持稳中求进工作总基调，以推进供给侧结构性改革为主线，加快五大产业发展，深化项目引领、创新驱动，坚决做好稳增长、促改革、调结构、惠民生、防风险各项工作，努力实现经济社会平稳健康发展。

　　遵循市“十三五”规划，衔接全面建成小康社会目标，对标与全国经济发展同步，2017年我市主要经济指标初步安排为：地区生产总值增长6.5%，规上工业增加值增长8%，固定资产投资增长12%，一般公共预算收入增长6%，社会消费品零售总额增长9%，出口创汇增长6%，实际直接利用外资增长15%以上，城镇和农村常住居民人均可支配收入增长与经济发展同步。

　　各位代表，我们正在步入振兴发展的新阶段，机遇与挑战同在，希望与压力共存，需要我们坚定信心全力推动振兴发展。我们的信心来自于以习近平同志为核心的党中央对东北振兴的重视与关心，省委、省政府的关怀与支持，一系列重大政策举措助推我市加快发展；我们的信心来自于市委的正确领导、人大政协强有力的支持以及四大班子精诚团结，为我们营造风清气正的干事创业氛围；我们的信心还来自于本钢与地方同心共进、生物医药新兴产业不断壮大、一批重大项目加快建设、民生持续改善，特别是全市人民的实干创新与真诚奉献，为振兴发展注入了强大动力。有信心就有希望，有信心就有力量，本本分分做人、点点滴滴做事的本溪精神时刻激励着我们奋发进取、攻坚克难。我们坚信，只要始终坚持发展第一要务，坚持一张好的蓝图真正绘到底，一任接着一任干，就一定能够开创老工业基地振兴发展的新局面。

　　2017年，市政府将做好以下工作：

　　（一）抓发展促进稳增长，振兴实体经济培育新动能

　　抓五大产业，增强发展后劲。集中力量发展五大产业，做强实体经济。以“大生态”产业引领绿色发展，推动生态产业链条集聚。以“大旅游”产业蓬勃发展进一步提升枫叶之都品牌形象，加快经济结构、产业结构调整。以“大健康”产业推进一二三产业联动融合发展，提升中国药都竞争力。以“大材料”产业支撑壮大绿色钢都实力，推动工业结构优化升级。以“大数据”产业创新发展带动大数据应用，提升信息化发展水平。

　　抓存量，提实力。一是加快规上工业企业发展。精准帮扶157户规上工业企业稳定运行。因企施策、一企一策，重点解决中小微企业资金、技术、人才等方面难题，积极引导中小微企业参与“双创”，扶持成长型企业加快发展，使 66户产值在1000万元以上的规下企业快速提升晋档、50户退出企业重回规上，实现规上工业企业数量增加、规上工业正常生产运行企业数量增加目标。二是促进产业结构优化。坚定信心推动生物医药新兴产业发展，上海绿谷、上药集团等一批重点项目竣工投产，发挥高新区引领沈本一体化融合发展先导优势，启动中德生物技术和医药产业合作园区建设，产业结构调整实现新突破。改造提升冶金支柱产业、钢铁深加工产业，推动本钢冷轧高强钢等重点项目达产达效。大力培育发展电子信息、消费品等产业。三是开拓工业产品市场。深化以企业为主导的产学研用创新体系建设，发挥高新区示范作用，鼓励高校科技成果转化，加强高科技、高附加值产品研发，强化品牌建设，提高产品市场占有率。推进辽宁九洲龙跃、华岳精工等14户企业的20种产品纳入省推广目录。四是推进制造强市建设。推动冶金、装备制造、建材等行业企业信息化建设，提高企业自动化、智能化和网络化水平，引领“两化”深度融合。五是强化人才建设。引进、培育优秀企业家、管理人才以及工程技术人员，拓展工匠培养平台，让更多的大工匠成为推动“本溪制造”崛起的主力军。

　　抓增量，上项目。一是坚定不移开展招商引资。围绕五大产业发展，坚决“走出去”“请进来”，全方位开展链条式招商引资。发挥板材、曲轴等原材料及深加工优势，争取引进比亚迪轨道交通生产基地等项目，争取打造完整的新能源汽车产业链。加快天士力大健康示范社区等在谈项目尽快签约落地，进一步构建完整的大健康产业链。继续围绕本钢上下游供应商、客户搭建招商平台，引进配套项目，延伸钢铁深加工产业链。争取引进大国企、大民企落户本溪。加快落实与江苏省在项目、产业发展等领域的全方位对接合作，引进储备一批牵动性强、科技含量高的产业项目。二是加快项目开复工。调整投资结构，扩大民间投资，千方百计增加有效投入，继续滚动推进200个重点项目。健全并联审批等服务机制，有效解决项目建设难题，4月份、8月份组织开展两次项目拉练检查活动，推动重点项目特别是一批新项目顺利开复工。加速推进投资58亿元的思山岭铁矿、投资30亿元的天士力东北现代中药示范工厂、投资10亿元的上海绿谷产业园一期、投资10亿元的辽宁祥云健康产业园、投资18.7亿元的观音阁水库输水工程及南芬供水工程等在建项目建设，确保早日竣工达产达效。全力推进投资15亿元的福耀高端浮法玻璃、投资10亿元的万达广场、投资18.6亿元的康力顺势产业园、投资10亿元的辽宁腾龙房车制造基地等项目开工建设。

　　抓消费增长，补服务业短板。一是促进旅游业快速发展。贯彻落实《旅游促进条例》，制定全域旅游示范区规划，强力推进全域旅游示范区创建工作，促进旅游业从观光游向体验式休闲度假游转型。加快旅游基础设施建设，争取关门山、五女山、枫林谷晋升国家5A级旅游景区，铁刹山、绿石谷晋升国家4A级旅游景区；加快自驾车、房车营地建设；市、县（区）出台扶持政策，支持新建一批特色精品民宿、特色旅游小镇，多层次打造旅游精品，提升旅游承载力。加强旅游商品市场开发。提高旅游公共服务水平，完善游客服务中心、旅游公厕、智慧旅游工程建设。强化旅游宣传营销，在央视、新浪网等媒体投放影视、城市宣传广告，提升城市知名度。以创建全域旅游示范区为引领，加快“多规合一”规划修编工作，促进旅游业与文化、健康、养老等产业融合发展，发挥旅游业对生态文明建设积极作用，带动经济结构转型升级。加快五女山等国有旅游景区改革步伐，巩固旅游资源整合上市基础。全市实现旅游总收入293亿元。二是推动商贸物流业转型升级。深入实施“互联网+流通”行动计划，实现线上线下融合发展。推动电子商务、商贸物流和会展业加快发展。加快农产品流通体系建设，实现农商互联。大力发展连锁经营等生活性服务业，推动形成便利快捷的社区商业圈。加快红星美凯龙等大型城市综合体和长江农副产品智能物流交易中心项目建设。规划建设钢材现货交易市场。继续开展丰富多彩的主题促销活动，打造12条特色夜市，推动“假日经济”“夜经济”持续活跃。

　　抓县域经济，强三农发展。推进农业产业结构、产品结构优化升级，加快县域经济发展、小城镇建设，努力实现农业更强、农民更富、农村更美目标。坚持打绿色牌、生态牌，大力发展特色高效农业，推进设施农业建设，加快种植业结构调整步伐，重点打造中药材、食用菌、山野菜、果业、优质米及观光农业六大特色产业经济区。实施北药产业规划，建立中药材标准化种植示范村7个。积极组织桓仁山参等申报国家生态原产地保护产品。促进农村一二三产业融合发展，深化农业农村产权制度改革，加快培育新型农业经营体系，全力推进桓仁省级农产品加工示范集聚区建设，信息进村入户工程实现全覆盖。加大金融机构扶持农村企业发展力度。抓好玻璃制品、葡萄酒等县域特色工业园区建设，发挥优势潜力，打造发展强力支撑。加快宜居乡村建设，完善建设小城镇，培育一批旅游、商贸、制造等特色小城镇，成为吸纳农村劳动力转移就业、创业首选地。

　　抓政策机遇，争发展先机。深入落实中央7号、国发28号及国发62号等文件政策举措，创新理念，积极争取国家产业衰退城市试点、国家可持续发展议程创新示范区、全域旅游示范区等一批含金量高的政策在我市落地生根。密切跟进生物医药集群新兴产业三年行动计划、双元培育改革试点和东北振兴三年滚动计划等项目申报进展。全力实施溪湖区老工业区和南芬区独立工矿区搬迁改造，力争歪头山独立工矿区搬迁改造、田师付采煤沉陷区治理纳入国家支持范围。落实本溪县、桓仁县作为国家重点生态功能区的要求，结合实际科学制定两县产业准入负面清单。通过政策叠加红利加速释放，助推持续健康发展。

　　（二）促改革激发新活力，进一步破解体制机制障碍

　　抓改革，促开放。一是继续深入推进“三去一降一补”。去产能方面，支持企业通过技术改造、兼并重组等方式化解过剩产能。去库存方面，结合棚户区改造千方百计化解房地产库存，促进房地产市场良性发展，商品房销售面积增长12%。去杠杆方面，改进金融服务，大力推进PPP项目，以财政资金撬动社会资本、金融资金，投向重点领域项目建设。降成本方面，合理降低工商业用电和工业用气价格,进一步降低企业成本，引导企业提升管理水平。补短板方面，进一步补齐经济结构、产业结构、投资结构短板，培育经济增长新引擎。二是深入推进“放管服”改革。以清单管理推动减权放权，完善市政府部门权力清单和责任清单。完善事中事后监管制度。继续整合简化审批程序，明确办结时限，最大程度方便群众和企业。健全涉企行政执法备案登记和行政处罚备案审核制度。三是推动行政管理体制改革。进一步优化市政府机构设置，减少管理层级，强化机构编制管理，行政事业编制实行总量控制、自然减员，提高行政效率。政府公务员和事业单位财政供养人员一律只减不增、内设机构一律不增加。市直党政群机关再精减编制5%，市直事业单位再精减编制10%。全面加强城市管理，调整城市管理体制，按照“属地管理、重心下移”原则，理顺市、县（区）职责，构建全覆盖网格化管理格局。扩大县（区）管理权限，真正为县（区）松绑，释放县域经济、城区经济活力。四是稳步实施事业单位分类改革。加快推进经营性事业单位转企改制、公益性事业单位优化整合，全面清理承担行政职能事业单位的职能职责，稳妥推进行政类事业单位改革。五是深化推进国资国企改革。进一步规范市属控股公司法人治理结构，稳妥推进控股公司股权多元化。推进市直部门与全民企业脱钩并实施公司制改制。基本完成央企等国有企业“三供一业”分离移交任务。一企一策推动厂办大集体改革取得实质性进展。同时，稳步推进农业、林业、教育、卫生等重点领域改革。六是促进出口增长。全力支持本钢开拓“一带一路”等国家市场，扩大新兴市场产能输出合作。推进综合保税仓库和跨境电商业务发展。抢抓辽宁自贸区试点机遇，与营口港合作建设陆港，建成陆海空联运紧密衔接的对外开放体系。

　　抓融资，破难题。一是用足国家对符合条件的东北企业申请上市给予优先支持的利好政策，重点推动旅游控股公司、高新区重点企业等实现上市融资，打造一批科技领先、高成长性的上市公司。做好上市企业储备、培育、申报工作。二是继续推进设立五大产业投资基金，积极与国开行、农发行在棚改、专项建设基金、政府购买服务等领域深入合作，为项目建设提供资金保障。三是加快推进地方银行机构改革，支持市商业银行升级为本溪银行、农信社全面改制为本溪市农村商业银行，激发地方金融发展活力。

　　抓服务，优环境。一是全力服务本钢发展。继续推进解决制约本钢发展问题，本地采选企业与本钢签订铁精粉供货协议量力争突破500万吨。进一步搭建本钢与地方企业配套对接平台，拓展合作空间。二是提升服务企业水平。总结推广高新区驻区企业和高校评议市政府涉企部门服务经验，在全市范围内筛选100户规上平稳运行企业、50户停产半停产企业、50户成长型企业和2017年新开工、在建项目，开展对市政府部门服务评议工作，以服务成效评估倒逼政府工作效能提升、工作效率提高。三是打造良好的营商环境。落实《辽宁省优化营商环境条例》，成立营商环境服务机构，市、县（区）形成合力共同打造企业发展良好环境。完善金融服务体系，健全银政企对接机制，组建融资担保公司、民营企业发展基金，切实解决民营企业特别是中小微企业融资难、融资贵问题，新增社会融资220亿元以上。强化人才保障服务，调动科研院所和高校科技人员积极性、创造性，促进科技成果转化，推动大众创业、万众创新，力争打造全省最好的营商环境。

　　（三）防风险守住发展底线，进一步巩固企稳向好发展基础

　　抓社保基金支付风险防范，确保按时足额发放。继续深入贯彻落实省委19号文件精神，进一步加强企业养老保险基金收支管理，多方筹集资金，确保退休人员养老金按时足额发放。同时，积极做好医疗、工伤保险基金风险防控，承担好国家、省养老、医疗保险改革任务。

　　抓政府债务风险防范，确保总体可控。将政府债务风险防范化解作为硬性指标纳入对各县（区）、市直各责任部门绩效考评内容。坚决遵循“借用还”一体化原则，落实债务责任。严格执行限额管理，有效控制债务规模，规范举债行为。制定可行措施，防止政府债务风险升级。通过大力推进国资国企改革和事业单位改革、PPP项目、加快产业发展等措施，化解债务总量，确保不发生政府债务风险。

　　抓财政支出风险防范，确保财政平稳运行。通过改革、盘活、挖潜增加财力。抓好财政增收及年度预算编制工作。进一步做好拓宽财源渠道、加强税收征管、合理处置资产等工作。全面清理、核定项目支出，削减和压缩不必要支出，坚决完成“保工资、保运转、保民生”任务。

　　抓社会稳定风险防范，确保社会和谐发展。深入开展平安本溪建设，加快推进立体化社会治安防控体系建设，提升全社会整体防控效能。开展常态化打击整治治安问题等专项行动，抓好安全生产、食品药品安全等工作，坚决防止重特大治安灾害事故发生。加快推进信访工作法治化建设，全力做好党的十九大等重大活动安保工作。继续全面深化公安改革，推出一批便民利民新举措。全面实施“七五”普法依法治市规划，推进公共法律服务体系建设。巩固全国双拥模范城成果，构建和谐的军政军民军地关系。

　　（四）惠民生提升获得感，进一步推动经济社会持续健康发展

　　抓民生改善，增强群众幸福感。继续为百姓办好十项民生实事。一是大力实施主城区环境综合整治。落实综合整治实施方案，坚决完成承诺的2017年任务目标，实施210个住宅区环境综合整治，“暖房子”工程333.25万平方米，整治街巷路146条，拆除居民小区和道路两侧违建乱建5070余处，强化物业管理；通过开展道路交通管理等措施，缓解城市交通拥堵。建立城市管理长效机制，巩固主城区环境综合治理、新区建设成果。推行垃圾焚烧发电及环卫一体化等新举措，促进国家卫生城市、全国文明城市加快创建。二是大力实施精准扶贫。继续落实“六个精准”和“五个一批”要求，扶持发展生态农业、特色养殖、休闲旅游等优势产业，实现桓仁贫困县摘帽、30个贫困村销号、11650名贫困人口脱贫。三是始终保持就业稳定。强化就业困难群体援助，保持全市零就业家庭动态为零，做好离校未就业大学生就业服务，确保有就业意愿的困难家庭高校毕业生100%兜底安置就业。城镇新增就业2.3万人，登记失业率控制在4.5%以内。四是实施棚户区改造8392套，加大棚改货币化安置力度。新增低收入住房保障对象租赁补贴100户，筹集公租房实物房源200套。五是完成平山区、明山区义务教育均衡发展省检任务，加快太子河新城实验学校项目建设。六是按照“大专科、小综合”模式，加快市中心医院太子河新城综合病房楼项目建设并投入运营。七是不断提高社会保障水平，健全特困人员救助供养制度，惠及5000人。八是为贫困残疾人适配辅助器具2000件，完成500户贫困残疾人家庭无障碍改造。九是加强食品安全监管，积极创建国家食品安全放心城市，本溪县全面通过食品安全放心县评价验收。十是提高养老服务水平，市失能半失能老人康复养护中心投入运营，大力发展居家和社区养老服务，吸引社会力量建设一批养老机构。

　　抓公共服务，推动社会事业全面进步。一是促进教育事业稳步发展。推进学前教育优质普惠广覆盖，启动实施第三个学前教育三年行动计划。推进义务教育优质、均衡发展，继续完善义务教育联盟体制机制。推进普通高中教育特色化、国际化发展，深化普通高中课程改革，加快东方剑桥本溪国际学校项目建设。优化职业教育布局，组建职教集团，落实双元培育试点工作，推进产教研用深度融合。大力引进民间资本进入教育领域，完善落实民办教育发展扶持政策。推进辽宁中医药大学和辽宁医药职业学院尽快搬迁。加强师德师风建设，严厉查处在职教师有偿补课行为，打造高素质教师队伍。规范整顿校园周边“小餐桌”。二是推进卫生计生事业发展。建立强有力的领导体制和医疗、医保、医药“三医”联动工作机制，全面推进医保支付方式改革，建设现代医院管理制度和符合行业特点的人事薪酬制度。加快分级诊疗制度建设，健全社区和农村医疗服务体系。落实《中国（本溪）大健康产业规划（2016-2020）》，推进健康本溪建设。全面落实计划生育各项政策。三是加快文化体育事业发展。强力推进文化强市建设。启动市人民文化宫舞台改扩建工程。打造现代新华书城。启动本溪湖工业遗产群一号高炉加固修缮工程。建设辽宁东北义勇军博物馆、桓仁满族民俗文化园。举办全市群众文化节，实现大型警示教育评剧《中秋泪》全省巡演，推动大型音舞诗画《映像本溪》进入旅游市场。继续开展全民健身运动。加强城市公园、步道体育公共设施建设。承办好2016-2017赛季CBA联赛。启动国家级航空飞行营地示范工程、“北方陈家沟”太极养生谷项目建设。

　　抓基础设施建设，增强发展支撑。一是加快城市基础设施建设。协调推进辽中环高速本溪段等在建项目加快实施，加快桓仁抽水蓄能电站、沈丹线及溪桓铁路电气化改造、桓仁高铁、本桓高速、市区绕城公路等项目前期工作，争取本桓高速等工程开工建设。维修改造解放北路、溪湖路、彩屯大桥等5条道路和桥梁。打造水清岸绿的太子河城市慢行系统。推进凤凰山等公园建设及规范管理。启动青云山综合规划开发。推动城市公交优先发展，加快公交基础设施建设，挖掘社会潜力布局建设立体停车场，强化停车管理，破解城市停车难题。二是加快推进新城建设。完善沈本新城生活服务设施配套，推进张仲景大街建设，打通木兰路与沈本产业大道的交通连线，形成日月岛片区环路。深入推进太子河新城新型城镇化一期、“新溪湖·印象”开发建设，推动威宁大街等地下综合管廊建设，鲍家两洞桥项目实现竣工通车。三是继续推进智慧城市建设。智慧山水（本溪）运行管理中心建成运营。推进“互联网+政务服务”示范工程建设并申请“新型智慧城市”试点，在智慧消防、智慧停车等领域实现新突破，智慧公交全面迈进“刷卡”时代。

　　抓生态环境建设，提升发展效益。树立绿水青山就是金山银山的理念，坚持严格保护、合理开发，推动生态文明建设取得积极进展。落实《本溪市环境总体规划（2015-2030年）》，推动重点行业污染治理设施提标改造，淘汰落后产能，严格执行无组织排放整治，深化农村面源污染整治。继续实施“青山、碧水、蓝天、沃土”工程。保护和完善我市特有的森林生态系统。拆除小锅炉241台，淘汰黄标车及老旧车辆2449台，空气质量优良天数比例达到80%。科学调整水环境功能区规划，在实施桓仁水库、观音阁水库生态环境保护工程的同时，处理好保护与发展的关系，为工业、农业、旅游等服务业发展留出空间。公开饮用水水源水质信息，强化主城区污水处理。实施城市山洪沟治理。完成我市土壤环境质量国控监测点位设置和监测网络建设。划定生态保护红线，推进规划环评改革。完善监测体系，提高环境预警分析能力，严格环境执法网格化监管，强化落实《环保法》四个配套办法。完成创卫、创模、创建生态文明建设示范市阶段性目标。

　　（五）强自身树立良好形象，为振兴发展提供坚强保障

　　我们将倍加珍惜历届市委、市政府创造的良好政治生态，在市委的坚强领导下，全面抓好重点工作任务落实，努力建设法治政府、诚信政府、服务政府、廉洁政府。一是坚持依法行政。自觉接受市人大及其常委会的依法监督和市政协的民主监督，认真办理人大代表建议和政协提案。自觉接受社会舆论和新闻媒体监督，加强行政执法监督，规范行政执法行为。二是坚持担当实干。不折不扣贯彻省委、省政府和市委的决策部署，坚决履行好为人民服务的光荣职责，敢于直面改革发展热点、难点问题，敢于解决政府系统不作为、慢作为、乱作为突出问题，坚持干字当先，想干事、敢干事，以钉钉子的精神，盯住重点项目、重点工作，一抓到底、抓出成效，不断开创振兴发展新局面。三是坚持廉洁从政。深入贯彻党的十八届六中全会精神，牢固树立“四个意识”，承担起全面从严治党的主体责任，认真贯彻执行《条例》和《准则》，深入推进党风廉洁建设，认真履行“一岗双责”，强化考核督查问责，始终维护本溪风清气正的政治生态，让企业更好发展、项目加快建设，实现经济社会持续健康发展。

　　各位代表，我们站在了全面振兴的新起点上，砥砺奋进，重任在肩。让我们在市委的坚强领导下，坚定信心，振奋精神，攻坚克难，努力开创美丽本溪、健康本溪、幸福本溪建设新局面，以优异成绩迎接党的十九大胜利召开！
